# Across the Social Network of the Gut: Bacterial, Fungal, and Viral Determinants of Checkpoint Inhibitor Efficacy and Toxicity

**DOI:** 10.3390/ijms27062538

**Published:** 2026-03-10

**Authors:** Andreea Laura Antohi, Andreea Daria Gheorghiță, Octavian Andronic, Gratiela Gradisteanu Pircalabioru, Andreea-Ramona Treteanu

**Affiliations:** 1 Faculty of Medicine, Grigore T. Popa University of Medicine and Pharmacy, 16 Universitatii Street, 700115 Iasi, Romania; antohi.andreea31@yahoo.com (A.L.A.); andreea.gheorghita05@gmail.com (A.D.G.); 2 Faculty of Medicine, Carol Davila University of Medicine and Pharmacy, 37 Dionisie Lupu Street, 020021 Bucharest, Romania; octavian.andronic@umfcd.ro (O.A.); andreea-ramona.treteanu0720@stud.umfcd.ro (A.-R.T.); 3 Innovation and eHealth Center, Carol Davila University of Medicine and Pharmacy, 37 Dionisie Lupu Street, 020021 Bucharest, Romania; 4 Department of Botany and Microbiology, Faculty of Biology, University of Bucharest, 030018 Bucharest, Romania; 5 Research Institute of University of Bucharest (ICUB), University of Bucharest, 050095 Bucharest, Romania; 6 eBio-Hub Centre of Excellence in Bioengineering, National University of Science and Technology Politehnica Bucharest, 060042 Bucharest, Romania

**Keywords:** mycobiome, virome, gut microbiome, immune checkpoint inhibitor, fecal microbiota transplant, cancer

## Abstract

Recent findings suggest that the gut microbiome significantly influences cancer outcomes, including responses to immune checkpoint inhibitor (ICI) treatments. Although early research focused on gut bacteria, it is now understood that the microbiome includes a bacteriome, virome, and mycobiome, all of which can modulate host immunity. Some commensal bacteria enhance anti-tumor immune responses and improve ICI efficacy, as demonstrated in both mice and patients. Fecal microbiota transplants (FMT) from patients responding to ICI have successfully reversed resistance in certain non-responders. In addition to bacteria, gut fungi and viruses are gaining attention as further factors influencing ICI effectiveness and toxicity. Recent multi-omics studies across cancer cohorts show that fungal and viral populations in the gut vary between ICI responders and non-responders. Commensal fungi may shape anti-cancer immunity by inducing inflammatory or tolerogenic pathways, while viral components can stimulate innate immune sensors that promote tumor surveillance. On the other hand, gut dysbiosis marked by expansion of pathobionts (including opportunistic fungi) and reduction in beneficial microbes is linked to serious immune-related adverse events (irAEs) such as ICI-induced colitis. This review discusses the multi-kingdom gut microbiome–bacteria, fungi, and viruses–and their interactions with the immune system in cancer therapy. We emphasize known mechanisms linking these microbes to anti-tumor immunity, overview human studies associating gut microbiome profiles with ICI outcomes and explore strategies to modulate the microbiome to enhance ICI efficacy while reducing toxicity. Understanding and utilizing the gut mycobiome and virome in conjunction with the bacteriome could pave the way for new biomarkers and therapeutic adjuvants in cancer immunotherapy.

## 1. Introduction

Cancer continues to be a significant global health challenge. The most recent GLOBOCAN report estimates approximately 20 million new cancer cases and 9.7 million deaths in 2022, with around 53.5 million people living five years post-diagnosis; roughly one in five people will face a cancer diagnosis in their lifetime [1]. These numbers highlight the magnitude of the challenge and the necessity for improved, more tolerable therapies. In the United States, around 2,041,910 new cancer cases and 618,120 deaths are expected in 2025 [2]. Although mortality rates have consistently declined through 2022—preventing nearly 4.5 million deaths since 1991 through smoking reduction, earlier diagnosis, and improved treatment—substantial disparities remain [2].

Over the past decade, immuno-oncology has redefined clinical practice by utilizing the host’s immune system instead of directly targeting or starving tumor cells [3,4,5,6]. Therapeutic classes currently include: (i) immune-checkpoint blockade against PD-1/PD-L1 and CTLA-4, with emerging pathways like LAG-3 becoming standard practice; (ii) adoptive cell therapies—CAR-T for hematologic malignancies and TILs/CAR-NK moving into solid tumors; (iii) therapeutic vaccination, including custom neoantigen and dendritic-cell approaches; (iv) oncolytic virotherapy (T-VEC); (v) cytokine/immune-modulating regimens [3,7,8,9,10]. Mechanistically, these approaches are complementary—disabling inhibitory circuits, providing tumor-reactive effector lymphocytes, priming de novo responses and readjusting the immune system environment [9]. Still, lasting benefits are heterogeneous, and toxicity can be substantial, indicating tumor-intrinsic and microenvironmental resistance mechanisms. Consequently, biomarker-driven combinations and improvements in delivery and formulation represent active frontiers [5,11,12].

Immune checkpoint inhibitors (ICIs)–monoclonal antibodies that restore anti-tumor T cell activity by blocking inhibitory receptors like PD-1, PD-L1, CTLA-4–have revolutionized the management of many malignancies [6]. However, only a subset of patients attains lasting remissions with ICIs, and others face considerable immune-related toxicities [6,13,14]. This variability has led scientists to explore factors beyond tumor genetics that influence ICI outcomes, with the gut microbiome emerging as a key player [15,16]. The human gastrointestinal tract contains a vast and diverse microbial ecosystem, collectively referred to as the gut microbiota [17]. This intricate community of microorganisms has developed alongside the host and is essential for digestion, metabolism, and particularly in shaping the development and function of the immune system [17,18,19]. In recent years, cancer researchers have grown more aware that gut microbes can influence both cancer progression and the efficacy of cancer therapies [15,16].

Early studies in 2015 provided proof-of-concept that gut bacteria can influence cancer immunotherapy [20,21]. In mouse models, the depletion of commensals through antibiotics was shown to reduce the anti-tumor effectiveness of ICIs [15,20]. On the other hand, transferring gut microbiota from one animal to another could convey the level of ICI responsiveness [15,21]. Around the same time, divergent findings in mice from different facilities highlighted that variations in gut flora might account for inconsistencies in ICI treatment outcomes [15,20,21]. These discoveries ignited a wave of research on particular bacterial strains that enhance anti-cancer immunity. Certainly, both preclinical and clinical studies soon confirmed that patients who respond to ICIs possess a distinct gut microbial composition compared to non-responders [15,22,23,24]. Specific bacteria, like *Bifidobacterium*, *Akkermansia*, *Faecalibacterium*, *Bacteroides* spp., have consistently been linked to positive ICI responses across multiple cancers [15,23,24,25]. Notably, fecal microbiota transplants from ICI-responsive donors demonstrated the capacity to overcome resistance in some refractory patients [26,27,28], indicating a causal relationship between the gut microbiota and ICI effectiveness [15]. Additional studies showed that baseline microbiota characteristics could also anticipate susceptibility to ICI-induced toxicities such as colitis [29,30], suggesting the gut environment affects both the effectiveness of treatment and safety.

Initially centered on gut bacteria, the gut ecosystem also consists of commensal fungi–the gut mycobiome, viruses–gut virome, archaea and protists [17]. These additional microorganisms, while less abundant than bacteria in terms of biomass, can produce immunologically active molecules and interact with both the host and bacterial communities significantly. The roles of the virome and mycobiome in cancer and immunotherapy have been largely overlooked and thus remain unclear. Nonetheless, advanced metagenomic and multi-kingdom studies are beginning to reveal how fungi and viruses can also influence anti-tumor immunity and ICI outcomes [31,32,33]. Since ICIs activate the immune system broadly, it is likely that the entire gut microbiome, across multiple kingdoms, affects the balance of immunity and tolerance throughout treatment.

Despite the growing body of literature, the field remains heavily focused on isolated bacterial associations. To address this, our review moves beyond a bacteria-centered descriptive approach to advance a multi-kingdom integrative perspective. We examine bacteria, fungi, and viruses not as isolated entities but as components of a dynamic ecological network shaping systemic immune tone. By comparing mechanistic pathways across microbial kingdoms and identifying areas of convergence, such as innate immune receptor activation, metabolite signaling, and interferon regulation, we aim to provide a more unified conceptual framework. Furthermore, we highlight underreported aspects of cross-kingdom interactions and propose testable hypotheses to guide future translational research.

To maintain conceptual clarity within this broad and rapidly evolving field, this review addresses three central questions:

1. How do representatives of different microbial kingdoms (bacteria, fungi, and viruses) modulate the efficacy of immune checkpoint inhibitors?
2. What mechanisms link multi-kingdom microbiome composition to immune-related adverse events (irAEs)?
3. Can multi-kingdom microbiome modulation strategies be rationally translated into therapeutic interventions that enhance efficacy while minimizing toxicity?

Rather than focusing solely on bacterial associations, we adopt a multi-kingdom framework that integrates bacteriome, mycobiome, and virome interactions and highlights their potential synergistic or antagonistic effects on systemic anti-tumor immunity.

Given the heterogeneity of study designs in the microbiome–ICI field, it is important to distinguish between levels of evidence supporting individual claims. Throughout this review, mechanistic conclusions derived from controlled preclinical models are distinguished from associative findings in observational cohorts, and from interventional human data obtained in early-phase clinical trials. While animal models provide important causal insights, translation to human immunotherapy requires validation in prospective clinical studies. Many currently reported microbial signatures remain correlative and should be interpreted cautiously until confirmed in adequately powered interventional settings. Clarifying this hierarchy of evidence is essential for responsible translation of microbiome research into clinical practice.

## 2. Literature Search Strategy

For this narrative review, we have gathered information from the specialized literature published in English over the past decade, with a focus on the interaction between the gut microbiome and cancer immunotherapy, particularly emphasizing the gut bacteriome, gut virome, and gut fungi. Databases such as PubMed, Scopus, and Web of Science were searched using combinations of the following keywords: “gut microbiome”, “gut microbiota”, “gut bacteriome”, “gut virome”, “gut mycobiome”, “cancer immunotherapy”, and “immune checkpoint inhibitors”. The types of articles included are reviews (narrative and systematic), preclinical and clinical studies, as well as meta-analyses, all of which were selected based on their title and abstract, particularly those examining the relationship between gut microbiota components and cancer immunotherapy. Exclusion criteria included non-peer-reviewed sources, conference abstracts, and studies not directly related to immunotherapy.

## 3. Immune Checkpoint Blockade: Mechanism and Clinical Relevance

The immune surveillance hypothesis suggested that our immune system patrols for and eliminates new cancer cells, preventing tumor development [34]. Both the innate and adaptive branches of the immune system collaborate to identify and remove emerging tumors [35]. Innate immune cells like natural killer (NK) cells, macrophages, and dendritic cells form an initial barrier of defense by killing abnormal cells directly or by presenting tumor antigens to T cells [36]. Adaptive immune cells, particularly cytotoxic T lymphocytes (CTLs), can recognize tumor-derived antigens and launch a precise attack on cancerous cells [36]. In healthy immune surveillance, these components collaborate to prevent malignancies [37]. Despite this, any cancer that arises in a host has, by definition, successfully managed to evade or suppress the immune response that would typically eliminate it [37]. Tumors achieve this immune evasion via various mechanisms, such as creating an immunosuppressive tumor microenvironment and utilizing regulatory “brakes” of the immune system referred to as immune checkpoints [4].

**Figure 1:**
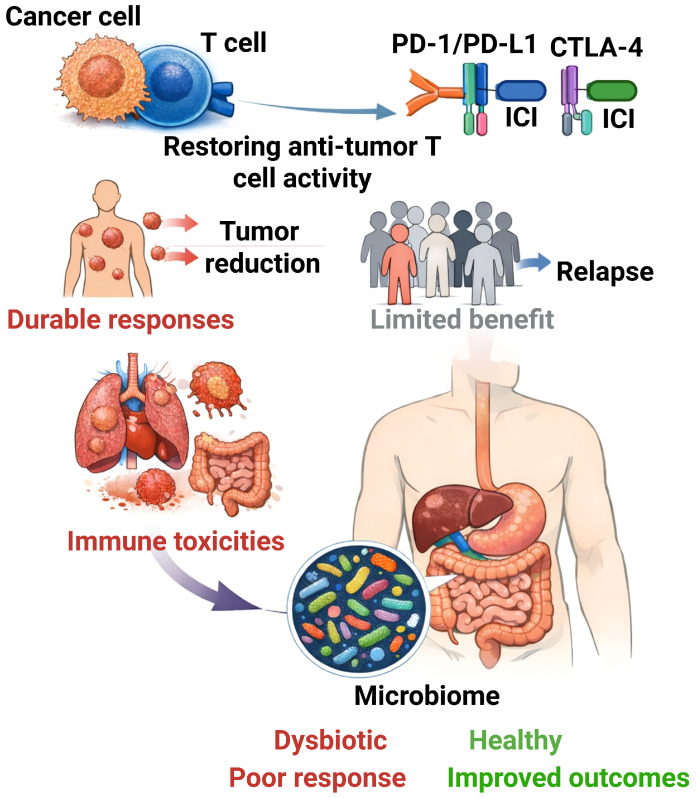
Immune checkpoint inhibitors and the role of the gut microbiome in cancer therapy. Immune checkpoint inhibitors (ICIs) enhance anti-tumor T-cell activity by blocking PD-1/PD-L1 and CTLA-4 pathways. Although they can induce durable tumor regression in some patients, the overall benefit is limited and may be accompanied by immune-related toxicities. The gut microbiome emerges as a key modulator of response and safety, with dysbiosis linked to poor outcomes and a healthy microbiome associated with improved therapeutic efficacy.

Immune checkpoints are inhibitory pathways embedded into the immune system that ensure self-tolerance and prevent excessive harm to healthy tissues during immune responses [38]. Cancer cells frequently exploit these checkpoints to turn off anti-tumor T cells, enabling the tumor to grow unchecked [38]. This understanding led to the idea behind developing immune checkpoint inhibitor (ICI) therapies–by blocking these inhibitory checkpoint molecules, we can unleash T cells and restore the immune system’s capacity to attack cancer [39,40,41,42]. In essence, checkpoint blockade therapy seeks to tip the balance in favor of immune activation rather than suppression, thus overcoming a primary escape mechanism used by cancer. Immune checkpoint inhibitors (ICIs) restore anti-tumor T-cell activity by blocking inhibitory receptors such as PD-1, PD-L1, and CTLA-4. Although these therapies have transformed oncology and achieved durable responses in multiple malignancies, clinical benefit remains limited to a subset of patients, and immune-related toxicities can be substantial. Host-related factors, including gut microbiome composition, have emerged as important modulators of both efficacy and safety (Figure 1).

ICIs have quickly transitioned from being experimental drugs to standard cancer treatments. They are currently approved for a broad range of malignancies, including melanoma, non-small cell lung cancer (NSCLC), renal cell carcinoma, bladder cancer, head and neck cancers, some colorectal cancers with mismatch-repair deficiency, Hodgkin lymphoma, among many others [43]. The clinical impact has been remarkable–for instance, advanced melanoma, once known to be fatal within a year, now has about half of patients surviving at 5 years in the era of combined checkpoint blockade [44]. Certain patients achieve full remissions that are sustained even after stopping treatment, suggesting the potential for cures in conditions once deemed uncurable [35]. Similar advancements have been observed in other cancers–for metastatic NSCLC, the addition of PD-1/PD-L1 inhibitors has greatly extended survival, with some patients achieving long-term remission [3]. Therefore, the expected outcomes of ICI therapy, although variable by cancer type, include a significant prolongation of survival for responders and a subset of long-term survivors that were not seen with previous therapies.

Nonetheless, despite these advancements, immune activation through CTLA-4 or PD-1 blockade by itself is not sufficient to manage tumor progression in all patients [4]. A significant number of patients either fail to respond to ICIs or show an initial response that is followed by a relapse [35]. Even in tumors with high immunogenicity, such as melanoma, where long-term response rates approach 60%, roughly 25% of patients exhibit primary resistance–characterized by disease progression within ~6 months of starting ICI–and many others later develop acquired resistance despite an initial response [44,45,46]. Tumor-intrinsic factors, tumor-extrinsic factors, and host factors (germline genetics, microbiome) all converge to influence outcomes. Significant efforts are made to identify reliable predictive biomarkers and to develop innovative approaches–whether new checkpoints, combination regimens, or personalized adjustments–to improve efficacy [43,47,48]. Patient-specific factors, such as genetic makeup and gut microbiota composition, have recently been linked to responsiveness to ICI therapy [31,49,50,51]. Research indicates that the gut microbiome can influence systemic immunity–certain commensal bacteria profiles in patients correlate with improved anti-PD-1 therapy outcomes, whereas antibiotic disruption of the microbiome is linked to poorer outcomes [43]. In mouse models, fecal microbiota transplant (FMT) from ICI-responding patients into non-responding mice was able to convey anti-tumor effects, showing that commensal microbes can affect treatment effectiveness [15]. The gut microbiome is a highly modifiable factor, raising the possibility of manipulating microbiota (through diet, probiotics, or FMT) to improve patient responses in the future [40].

ICIscan cause a variety of adverse effects, ranging from mild pharmacologic side effects to unique immune-related adverse events (irAEs) [4,6,52]. Typical general reactions include fatigue, rash, diarrhea, nausea, fever, pruritus, and musculoskeletal pain [39,53,54]. IrAEs can impact virtually any organ: skin and gut are commonly affected, followed by endocrine glands and liver, and less frequently the heart and lungs [55]. The profile of irAEs differs depending on the ICI target. CTLA-4 inhibitors often cause colitis, hypophysitis and dermatologic eruptions [56,57], whereas PD-1/PD-L1 inhibitors are more likely to lead to pneumonitis, thyroid dysfunction, arthralgias, and vitiligo [53,57]. Combination immunotherapy (CTLA-4 + PD-1 blockade) often increases toxicity for only a modest gain in efficacy [58]. In metastatic melanoma trials, adding a CTLA-4 antibody to PD-1 therapy roughly doubled the incidence of high-grade (≥3) irAEs compared with PD-1 alone [39,59,60]. Notably, thyroid immune-related events become more frequent with combined therapy [53,61,62]. These findings highlight that unleashing multiple immune “brakes” can produce synergistic anti-tumor effects at the cost of higher immune toxicity [4,39]. This baseline understanding of ICI toxicity provides a foundation for understanding how the host microbiome plays a role in further modulating these outcomes.

## 4. Microbiota: A General Overview

From birth, humans are colonized by a large community of microorganisms–the microbiota–especially on mucosal areas such as the gastrointestinal tract [17]. The gut microbiota is extremely diverse, containing thousands of species and multiple kingdoms of microbes that coexist in a symbiotic “superorganism” alongside the host [17,63]. Bacteria are predominant by cell count, with four major phyla (Firmicutes, Bacteroidetes, Actinobacteria, and Proteobacteria) representing approximately 98% of the microbial residents of the gut [64]. These bacteria are crucial for fermenting dietary fiber to produce short-chain fatty acids (SCFAs) that nourish colon cells, for synthesizing vitamins and aiding in the metabolism of bile acids and other compounds, and for colonization resistance against pathogens by outcompeting or inhibiting them [65]. Besides bacteria, the gut harbors smaller populations of fungi–the mycobiome, a virome of viruses, mainly bacteriophages that infect bacteria [64], archaea and protists, all being part of the gut ecosystem [65]. Although less abundant, these non-bacterial members can influence community dynamics–fungi can bloom when antibiotics wipe out competing bacteria [66], leading to issues like *Candida* overgrowth [67], while bacteriophages can modulate bacterial populations [68]. Overall, the gut microbiome functions as a complex, interdependent community, and sustaining its balanced state of eubiosis is crucial for health, whereas disruptions to its composition (dysbiosis) can negatively impact host physiology [69].

One of the most vital functions of the gut microbiota is its interaction with the immune system of the host [64]. The intestinal barrier–made of tightly joined epithelial cells, mucus secreted by goblet cells, antimicrobial peptides from Paneth cells and IgA from local plasma cells–serves as the primary line of defense that restricts microbes to the gut lumen [63,70]. It has become clear that the presence of commensal microbes is essential for proper immune development: germ-free animals have underdeveloped gut lymphoid tissue, low baseline IgA levels, and fewer effector and memory T cells [71]. The microbiota provides continuous stimuli (such as microbial DNA, lipopolysaccharides, and metabolites) that calibrate the host immune set-point–effectively adjusting the balance between an active immune response and immune tolerance [72].

Considering the profound immunological impact, it is not surprising that dysbiosis has been associated with multiple diseases [73,74]. External factors such as antibiotics, poor diet or infections can disturb the delicate microbial balance [75]. The loss of beneficial commensals or blooms of opportunistic organisms can disrupt the gut’s immune tolerance and barrier integrity [69]. Antibiotic-induced dysbiosis frequently increases gut permeability and allows microbial metabolites to enter the bloodstream, triggering chronic low-grade inflammation [64]. This type of systemic inflammation has been linked to metabolic disorders and a pro-tumorigenic environment [73]. Persistent dysbiosis is a known factor in conditions such as inflammatory bowel disease and colorectal cancer [73]. Patients diagnosed with colorectal cancer (CRC) have lower levels of butyrate-producing gut bacteria, indicating that diminished microbial production of anti-inflammatory metabolites such as butyrate may impair immune surveillance and promote tumor development [64,76]. Certain gut bacteria present in dysbiosis have been implicated in cancer risk: *Helicobacter pylori* infection can be a cause of gastric cancer [77]; some strains of *Escherichia coli* produce a toxin causing DNA damage in colonic cells [78,79]; and *Fusobacterium nucleatum*, which thrives in oral and gut dysbiosis, can be found in colorectal cancer tissue [80,81]. These insights have led to the microbiome being recognized as a factor in cancer’s “enabling characteristics” as a determinant of chronic inflammation, immune evasion, and even therapy resistance [78]. In summary, who inhabits our gut can shape our baseline cancer risk and tumor behavior.

Based on this, naturally, the gut microbiota has emerged as an important player in determining how patients respond to cancer therapies, particularly the latest class of immunotherapies [82]. Clinicians noted more than a century ago that severe infections could sometimes result in tumor regressions–an observation utilized by William Coley in the 1890s [83]. Coley purposefully injected cancer patients with combinations of live or inactivated bacteria, and at times observed remarkable tumor shrinkage accompanying the provoked fevers and immune activation. “Coley’s toxin” is recognized as the initial attempt of cancer immunotherapy and highlights the concept that microbial stimuli can activate the immune system better fight cancer [39,83,84]. In the contemporary age, current immune checkpoint inhibitors (ICIs)–drugs like anti-CTLA-4 or anti-PD-1/PD-L1 antibodies–have revolutionized oncology by achieving lasting remissions in a subset of patients. Nonetheless, a significant number of patients do not respond to ICIs, a fact that led to intense research into finding out why [35]. In 2015, two landmark studies first pointed to the gut microbiome as a possible key factor. Vetizou et al. (2015) showed that the anti-CTLA-4 antibody, ipilimumab, could only effectively manage tumors in mice that had certain *Bacteroides* species in their gut [20]. Germ-free or antibiotic-treated mice did not respond until *Bacteroides fragilis* was introduced [20]. However, Sivan et al. (2015) observed that the efficacy of anti-PD-L1 therapy in mice was increased by the presence of Bifidobacterium in the gut–mice from a colony with abundant *Bifidobacterium* had better tumor control compared to those lacking these bacteria and administering *Bifidobacterium* to the latter improved their outcomes [21]. Decades after Coley’s primitive bacterial adjuvant, we have circled back to understanding the powerful help that microbes can provide in the fight against cancer [83].

Considering that bacteria represent the largest and most studied part of the gut microbiome, it is in this area of research that most microbiome-oncology insights have been obtained. At this point, numerous preclinical and clinical studies have identified specific bacterial species that can boost or suppress anti-tumor immune responses. In this section, we outline key discoveries regarding the relationship between the gut bacteriome and the immune system in the context of cancer therapy, as well as the bacterial taxa associated with better or poorer results from ICIs.

### 4.1. Distinct Microbiota in Responders vs. Non-Responders

One of the first clues that gut bacteria influence immunotherapy outcomes came from comparing the stool of cancer patients who respond to ICIs and those who do not [85,86]. These studies frequently report microbial “signatures” linked to response [85]. In melanoma patients undergoing treatment with anti-PD-1 checkpoint inhibitors, responders had significantly greater gut microbial diversity and an abundance of *Clostridiales*/*Firmicutes taxa*, while non-responders often had microbiomes dominated by Bacteroidales [25,87,88]. Responders exhibited higher levels of *Faecalibacterium prausnitzii* and *Ruminococcus*, along with *Akkermansia*, whereas non-responders had more *Bacteroides*, which was linked with immunosuppressive cell populations in the blood [31,72,87]. In patients receiving anti-CTLA-4 treatment, small studies similarly indicate that those experiencing clinical improvements often had a gut microbiome enriched in Firmicutes, whereas those with poorer outcomes displayed an abundance of *Bacteroidetes phylum* bacteria [80,89,90]. Interestingly, it was found that *Bacteroides caccae* and *Bacteroides uniformis* were more prevalent in patients who responded positively to ICIs overall, implying that not all *Bacteroides* are “bad”–context matters [80,91,92]. However, a common trend observed in several studies is that pathobionts or dysbiotic indicators are often associated with non-response, whereas a diverse, commensal-rich ecosystem correlates with response [29,75,93]. These studies set the stage for exploring causality further.

Many specific bacterial taxa have been identified in various studies, and although we cannot cover them all here, some names appear repeatedly. On the “beneficial” side, many are well-established commensals known for their good metabolic or immunologic roles: *Akkermansia muciniphila* [94,95,96,97,98] (a mucus-degrading bacterium that can improve gut barrier integrity and stimulate mucosal DCs), *Faecalibacterium prausnitzii* [98,99] (an anti-inflammatory butyrate producer), *Bifidobacterium* species [99,100,101] (fiber-fermenters frequently used as probiotics), and members of the *Ruminococcaceae* [51,102] and *Lachnospiraceae* [51,103,104] families are consistently linked to better ICI responses [80]. Another notable genus is *Bacteroides*–although an excess of *Bacteroides* overall can indicate dysbiosis, specific species like *Bacteroides fragilis* and *Bacteroides thetaiotaomicron* have demonstrated enhancement of anti-CTLA-4 therapy in mice and were associated with beneficial immunity in some patients [20,86,105,106].

Conversely, scientists have identified bacteria that could be detrimental to cancer immunotherapy. These are usually organisms linked to pro-inflammatory dysbiosis or opportunistic infections [29,75,107]. An abundance of Proteobacteria is frequently seen as a warning sign in microbiome studies, as it may indicate an unbalanced state [91,102]. In patients treated with ICI, an overgrowth of Proteobacteria or certain species of *Enterococcus* and *Streptococcus* has been associated with poorer responses and increased immune-related side effects, such as colitis [72,102,106,108]. Bacteroidales members, despite containing some beneficial species, are often observed at higher levels in non-responders–possibly due to certain *Bacteroides* and *Prevotella* species that stimulate expansion of regulatory T cells and myeloid suppressor cells, blunting anti-tumor immunity [72,92,108,109]. *Bacteroides intestinalis* serves as an example of a potential pathogen, recently associated with severe checkpoint inhibitor colitis in melanoma patients [90,110]. Likewise, *Fusobacterium nucleatum*, known for promoting colorectal cancer growth and evading immune defenses, could hinder immunotherapy if present in the tumor microenvironment [78,81].

Several studies have associated gut microbiota composition with the likelihood of developing ICI-induced colitis, a typical irAE [93,111]. In patients with metastatic melanoma receiving ipilimumab, baseline microbial profiles were strikingly different between those who developed severe colitis and those who did not [30,90]. Notably, beneficial anaerobes in the phylum Bacteroidetes were enriched in patients who did not experience colitis, suggesting a protective effect of these commensals [39,90]. Members of the *Bacteroidaceae*, *Rikenellaceae*, and *Barnesiellaceae* families were more prevalent at baseline in patients who did not develop ipilimumab colitis [90]. These bacteria are thought to promote mucosal regulatory pathways [39]. Consistent with this, preclinical mouse studies showed that *Bacteroides fragilis* could protect against CTLA-4–induced colitis and was actually necessary for optimal anti-CTLA-4 tumor efficacy [20,106]. In contrast, patients who lacked these Bacteroidetes and instead had high baseline levels of Gram-positive Firmicutes were more susceptible to colitis. The initial abundance of the *Firmicutes phylum* (especially Ruminococcaceae family members like *Faecalibacterium*) correlated with an increased risk of ipilimumab-induced colitis [39,112]. Intriguingly, the same Firmicutes-rich profile was also linked with improved tumor response to treatment [39,112,113]. *Faecalibacterium prausnitzii*–a butyrate-producing anti-inflammatory bacterium–was found at higher levels in patients who had positive clinical outcomes, although these patients also had higher rates of colitis [30,114]. This indicates a double-edged sword: a microbiome that strongly boosts T-cell activity might enhance ICI efficacy at the expense of lowering the threshold for intestinal autoimmunity.

Beyond single cohorts, larger studies have strengthened the microbiome–toxicity link. A recent prospective study involving 195 patients receiving anti-PD-1 ± anti-CTLA-4 therapy found that baseline dysbiosis characterized by the growth of opportunistic bacteria was associated with subsequent severe irAEs [29]. Patients who later developed grade ≥ 3 toxicities had a significantly higher relative abundance of a prespecified “pathobiont” group at the start of the treatment, 8.2% compared to 4.8% in those without severe irAEs [29]. Moreover, when beginning ICI therapy, these patients showed an acute loss of beneficial *Ruminococcaceae* in their gut microbiome, a shift not seen in patients who tolerated treatment well [29]. By the time severe colitis or other irAEs manifested, levels of Ruminococcus had drastically decreased in the affected patients [29]. These findings suggest that a resilient, well-balanced microbiome that is rich in fiber-degrading and SCFA-producing bacteria may protect against immune-mediated toxicity, while a microbiome tilted toward pathobionts primes the host for unchecked inflammation. In practical terms, broad-spectrum antibiotic exposure has been associated with poorer ICI outcomes, including not only reduced efficacy but possibly higher risk of colitis according to some reports [24,91,97,115,116]. The lack of commensals like *Akkermansia muciniphila* and *Faecalibacterium* correlates with both poor tumor response and a higher incidence of gastrointestinal irAEs [29,108,111]. This highlights the integral role of the microbiome in adjusting the balance between the host immune system’s aggression and tolerance, also summarized in Table 1.

**Table 1:** Gut Bacterial taxa or features associated with ICI efficacy and toxicity.

Bacterial Taxon/Feature	Association with ICI Outcomes	Key References
*Akkermansia muciniphila*	↑ ICI efficacy (PD-1/PD-L1); absence → ↓ efficacy. Preclinical FMT restored response.	[24,29,32,94,95,96,97,98,99,102,108,117,118,119]
*Faecalibacterium prausnitzii*	↑ ICI efficacy (multi-cancer); SCFA producer. ↑ risk of CTLA-4–induced colitis (melanoma).	[30,31,32,39,98,99,106,114]
*Bacteroides* spp.	Context-dependent. Certain species (e.g., *B. fragilis*) → ↑ CTLA-4 efficacy and ↓ GI toxicity. Some Bacteroidales enrichment → ↓ PD-1 response.	[20,39,82,89,90,91,92,105,106,110,111,112,113,114,115,116,117,118,119,120,121,122,123,124]
*Bifidobacterium* spp.	↑ ICI efficacy (PD-1/PD-L1); enhanced DC activation and CD8^+^ response (preclinical + clinical). No strong irAE association.	[21,23,82,93,99,100,101,111,118,122,125]
High gut microbiota diversity	↑ ICI efficacy; ↓ risk of severe irAEs. Low diversity → ↓ response, ↑ toxicity risk.	[25,29,39,97,106]
Pathobiont enrichment	↓ ICI efficacy; ↑ severe irAEs (including colitis).	[29,116]

↑ = increased; ↓ = decreased.

It is important to understand that “good” and “bad” bacteria often rely on context. The overall impact of the microbiome on cancer immunity results from a complex balance: diversity and community structure likely influence outcomes as much as the presence or lack of any single species [80].

### 4.2. Why Do Microbiome Signatures Differ Across Studies?

Despite recurring associations between certain taxa and ICI response, reported microbial signatures vary substantially across studies. Several factors likely explain these discrepancies. First, differences in sequencing platforms (16S rRNA vs. shotgun metagenomics), taxonomic resolution, and bioinformatic pipelines limit cross-study comparability. Second, geographic location, dietary habits, antibiotic exposure, and baseline comorbidities shape microbiome composition independently of cancer therapy. Third, heterogeneity in cancer type, stage, and line of therapy introduces additional variability. Finally, many cohorts remain relatively small, increasing the risk of overfitting and limited reproducibility. These considerations underscore why simplistic “add one beneficial microbe” probiotic strategies may be insufficient and highlight the importance of ecosystem-level approaches.

### 4.3. Microbiome Signatures of Efficacy Versus Toxicity: Overlap and Divergence

Emerging data suggest that microbial configurations associated with improved ICI efficacy do not always align with those associated with protection from irAEs. In some cases, microbial signatures of response and toxicity partially overlap, whereas in others they appear to diverge.

Several taxa enriched in responders, particularly immunostimulatory, SCFA-producing commensals, have been linked to enhanced dendritic cell activation, Th1 polarization, and cytotoxic T-cell priming. While such immune activation is desirable for tumor control, it may simultaneously lower tolerance thresholds in mucosal tissues, increasing susceptibility to colitis or other irAEs.

Conversely, microbial communities enriched in regulatory or tolerogenic features may protect against toxicity but potentially attenuate anti-tumor immune responses. For example, taxa associated with increased Treg induction or anti-inflammatory cytokine production have been linked in some cohorts to reduced gastrointestinal toxicity, yet their impact on tumor response remains inconsistent.

Importantly, some microbial signatures appear to promote both efficacy and manageable immune activation without excessive toxicity, suggesting that the relationship is not purely antagonistic. This supports the concept of an immune activation continuum rather than a binary efficacy–toxicity dichotomy.

These observations have significant translational implications. Strategies aimed at indiscriminately enhancing immune stimulation through microbiome modulation may inadvertently increase the risk of irAEs. Future approaches should therefore aim to identify ecosystem-level configurations that optimize anti-tumor immunity while preserving peripheral tolerance.

### 4.4. Section Summary and Key Take-Home Messages

The gut bacteriome represents the most extensively studied microbial component influencing ICI outcomes. Evidence from preclinical models, observational cohorts, and early interventional studies consistently supports an association between microbial diversity, enrichment in SCFA-producing commensals, and improved therapeutic responses. However, certain taxa may simultaneously enhance anti-tumor immunity while increasing susceptibility to immune-mediated toxicity, illustrating the delicate balance between efficacy and autoimmunity. Importantly, bacterial signatures are context-dependent and vary across cancer types, geographic regions, and methodological platforms.

## 5. Gut Mycobiome in Immunity and Cancer

### 5.1. Composition and Roles in Health and Disease

The gut mycobiome represents a small but important portion of the microbiota. Fungal cells are significantly less numerous than bacteria, around of 0.1% or less of gut microbes, yet they include various yeasts and molds such as *Candida*, *Saccharomyces*, *Malassezia*, and others [32,85,126,127,128]. Under healthy circumstances, these fungi are present at low levels and might even provide benefits. Commensal fungi can aid digestion through fermenting dietary fibers and contribute to immune system development [129]. Interestingly, mice raised completely germ-free and fungus-free exhibit more significant immune deficiencies compared to germ-free mice that are colonized with a few fungi, suggesting that initial exposure to fungal antigens helps “train” the immune system [126]. Therefore, a balanced mycobiome likely aids in maintaining immune homeostasis, while disturbances in fungal populations are linked to disease. For instance, in inflammatory bowel disease (IBD) patients, the gut mycobiota frequently shifts toward increased levels of Candida and reduced diversity of harmless fungi like *Saccharomyces* [130]–changes believed to both partake in the cause of and exacerbate the chronic inflammation [126,131,132]. Another study found that *Candida tropicalis* was significantly increased in Crohn’s disease and formed abnormal biofilms alongside pathogenic bacteria on the gut lining [133], underlining how fungal and bacterial dysbiosis often go hand-in-hand [66].

Fungi have distinct cell wall components–β-glucans, mannans, chitin–that represent potent pathogen-associated molecular patterns (PAMPs) stimulating host immune response through pattern recognition receptors (PRRs) [134]. One key receptor is Dectin-1 (CLEC7A), a C-type lectin found on dendritic cells and macrophages that binds β-1,3-glucans selectively from fungal cell walls [135]. Engagement of Dectin-1 initiates intracellular signaling pathways that promote cytokine synthesis and facilitate the differentiation of Th17 and Th1 helper T cells [135,136], both critical for mucosal defense and integrity [137]. In such a manner, colonization with *Candida albicans* can trigger IL-17-producing T cells that protect against pathogenic bacteria invasion–a symbiotic immune activation that benefits the host [130]. However, if fungal growth is not kept in check, overactivation of the immune system may occur.

### 5.2. Fungi in Cancer and the “Tumor Mycobiome”

Recent research indicates that fungi are not confined to the gut lumen–they can spread or inhabit various tissues, including tumors [138,139,140,141]. Large-scale sequencing studies across thousands of human tumor samples have discovered detectable fungal DNA (and even whole cells microscopically) in many types of cancers, generally at low levels and with cancer-type-specific profiles [128,139,140]. One comprehensive study found that fungal signals were present in ~35% of samples across 17 cancer types, at an average of ~1 fungal cell per 10,000 tumor cells [128]. Intriguingly, the composition of these cancer-associated fungi varied by cancer: gastrointestinal (GI) tumors often had higher levels of *Candida* species, lung tumors had signatures of environmental molds like *Aspergillus* or *Blastomyces,* and pancreatic tumors displayed a notable predominance of *Malassezia* yeasts [128,140,142,143]. *Malassezia* is a genus normally present on skin and within the sebaceous glands, but researchers discovered it can migrate up the pancreatic ductal tree [140]. In a mouse model of pancreatic ductal adenocarcinoma (PDA), gut fungi migrated into the pancreas and significantly sped up the tumor progression [140]. PDA-bearing mice exhibited a ~3000-fold increase in fungal abundance in their tumors, with Malassezia predominating over other fungi. More interestingly, eliminating the fungi with antifungals protected the mice from tumor growth, while reintroducing Malassezia–but not Candida or other fungi–restored the cancer acceleration [140]. The mechanism was traced to the immune system’s complement cascade: Malassezia cell wall glycans triggered the mannose-binding lectin (MBL) pathway of complement activation, creating an inflammatory environment that promoted tumor progression [140]. Deletion of MBL or complement C3 eliminated this pro-tumor effect, implicating complement as a key mediator of fungus-driven oncogenesis [140]. In another study, Candida in colon tumors was correlated with increased metastatic disease and alterations in cell adhesion molecules, indicating that these fungi might promote a more aggressive tumor phenotype [128].

Conversely, it may be that some of these associations reflect fungi opportunistically colonizing immunosuppressed tumor environments rather than actively promoting cancer. Determining cause versus effect is a difficult challenge. Nonetheless, the emerging idea of a “tumor mycobiome” has important implications: fungi and their metabolites within tumor microenvironments might modulate local immune responses [138]. Fungi in tumors could continuously activate PRRs, leading to either tumor-promoting inflammation or anergic tolerance, depending on context. The tumor mycobiome also presents potential biomarker opportunities–a study found that the presence of specific fungi in tumor tissues was predictive of reduced patient survival, raising the possibility that fungal DNA detection could serve as a prognostic indicator [128]. This is a novel area of research, so mechanistic understanding is limited, but it is becoming increasingly clear that fungi are yet another important player in the complex ecosystem of the tumor microenvironment.

### 5.3. Mycobiome Influence on Cancer Immunotherapy (ICI Efficacy)

Given that gut bacteria can affect patients’ responses to immune checkpoint inhibitor (ICI) therapies, it naturally raises the question of whether the gut mycobiome also influences immunotherapy outcomes. Although research is in its incipient phases, initial findings suggest that the mycobiome indeed contributes to the variance in ICI efficacy as summarized in Table 2. A recent multi-cohort study analyzed 862 fecal metagenomes from cancer patients receiving ICI therapy across several cohorts to identify microbial characteristics linked to treatment response [32]. Strikingly, fungal species emerged as important indicators of response: a machine learning model using gut fungal profiles could predict ICI responders vs. non-responders with an average area under the ROC curve (AUC) of 0.87 [32]. This performance was equivalent to, and independent of, bacterial markers. In fact, the integration of fungal markers with bacterial biomarkers led to an enhanced predictive accuracy (AUC ~0.89), highlighting that the mycobiome offers supplementary Information beyond the bacteriome [32]. Specific fungi highlighted in that study include Schizosaccharomyces octosporus, a SCFA-producing fermentative yeast that was enriched in ICI responders’ guts [32]. SCFAs are known to strengthen anti-tumor immunity by enhancing T cell function [144,145], so this fungus might indirectly support the anti-cancer immune response through its metabolites. Another finding was that responders, as predicted by fungal markers, showed higher baseline levels of “exhausted” T cells in peripheral blood [32]. This could suggest that certain fungal components are causing a mild chronic T cell activation that primes the immune system. That alone could predict a more effective ICI outcome.

Additional clinical data comes from smaller cohort studies. In advanced hepatocellular carcinoma (HCC) patients receiving ICIs, one study reported that those who achieved lasting clinical benefit had a greater mycobiome α-diversity at baseline compared to non-responders [146]. This mirrored the pattern observed with bacterial diversity; however, the magnitude of differences in fungal communities was less pronounced than that of bacteria in this HCC cohort, and few specific fungal taxa showed significant association with outcomes [146]. Fungi may affect the response to immunotherapy primarily alongside bacteria instead of acting as the main contributors independently; interactions between the two, symbiotic or antagonistic, could modulate the net immunological outcome.

### 5.4. Fungal-Derived Immune Modulators (β-Glucans and Others)

Another way the mycobiome can affect cancer immunity is through isolated fungal components used as therapeutics. A notable example is β-glucan, the polysaccharide derived from yeast cell walls. In oncology, purified β-glucans, sourced from Saccharomyces cerevisiae or medicinal mushrooms, have shown immune-stimulating and tumor-suppressing properties [134,143,147,148]. Preclinical studies indicate that β-glucan can prime neutrophils and macrophages [136] to more effectively eliminate tumor cells, especially in the presence of tumor-targeting antibodies, resulting in an additional CR3-dependent cytotoxicity [147]. In the realm of checkpoint blockade immunotherapy, incorporating β-glucan has demonstrated promising synergistic outcomes in experiments [149,150,151]. A study conducted on mice [151] tested a particulate yeast β-glucan (WGP) together with anti–PD-1 and anti–PD-L1 antibodies. The combined treatment resulted in increased immune cell infiltration into tumors and a more favorable tumor microenvironment compared to administering ICIs alone–specifically, more dendritic cells and effector T cells accumulated in the tumor, while gene expression indicated a shift toward Th1/CTL responses [151]. Tumor growth was significantly slower in mice receiving the β-glucan + ICI combination, suggesting enhanced anti-tumor efficacy [151]. Mechanistically, it appears β-glucan stimulation can diminish the immunosuppressive activity of myeloid-derived suppressor cells and Tregs through Dectin-1 signaling, while also activating antigen-presenting cells [152]. The overall effect is to “unleash” the immune system further when PD-1/PD-L1 blockade removes inhibitory signals on T cells. Exploratory clinical reports suggested that β-glucan supplementation might help overcome resistance to PD-1 inhibitors in patients. In another study, cancer patients who had progressed on PD-1 inhibitors experienced tumor shrinkage or disease stabilization after starting a daily yeast β-glucan supplement alongside continued anti-PD-1 therapy [153]. Currently, clinical trials are beginning to formally test β-glucans as immunotherapy adjuvants [152,154].

**Table 2:** Gut Fungal findings associated with ICI outcomes.

Fungal Taxon/Feature	Association with ICI Outcomes	Key References
*Schizosaccharomyces octosporus*	↑ ICI efficacy (pan-cancer); enriched in responders; part of predictive fungal signature (AUC 0.87).	[32,143,155]
*Candida albicans*	Context-dependent. Moderate presence: neutral/unclear. Overgrowth → ↓ efficacy; associated with dysbiosis.	[32,143,156]
Core fungal response signature (n = 26)	Fungal profile predicted ICI response (AUC 0.87); multi-kingdom model (fungi + bacteria) ↑ predictive accuracy (AUC 0.89). Specific taxa enriched in responders vs. non-responders.	[32]
Saccharomyces paradoxus	↑ ICI efficacy (anti–PD-1 response).	[143]
Malassezia restricta	↓ ICI efficacy.	[143]
High fungal diversity	↑ ICI efficacy (modest effect size); no consistent ↑ irAE signal. Low diversity → microbiome instability.	[142,146]
Opportunistic fungal blooms (*Candida*, *Aspergillus*, *Pneumocystis*, *Fusarium*)	↑ irAEs (particularly under immunosuppression).	[157]

↑ = increased; ↓ = decreased.

### 5.5. Section Summary and Key Take-Home Messages

The gut mycobiome, though quantitatively minor, exerts immunologically significant effects through β-glucans and other fungal-derived molecules that engage innate immune receptors. Emerging multi-omics studies suggest that fungal signatures may independently predict ICI responsiveness and complement bacterial biomarkers. However, current evidence remains largely associative, and mechanistic validation is still limited. The concept of a tumor-associated mycobiome further expands the potential influence of fungi beyond the intestinal compartment.

## 6. Gut Virome in Immunity and Cancer

### 6.1. Composition of the Gut Virome and Baseline Role

The gut virome refers to the collection of viruses found in the gastrointestinal tract [158]. This includes bacteriophages as well as viruses that infect human cells [159]. Phages are believed to be highly prevalent in the gut–estimates indicate that viral particles might equal or exceed bacterial cells in feces [160,161]. Most of these are double-stranded DNA phages, members of families like Caudovirales and single-stranded DNA (ssDNA) phages of the family Microviridae, which infect common gut bacteria [161]. Through infection and lysis cycles, phages regulate bacterial population size and turnover by reducing the numbers of commensal bacteria that have grown excessively dominant [160,162]. Phages also mediate horizontal gene transfer between bacteria by transduction, some improving bacterial fitness or virulence, such as toxin genes or antibiotic resistance factors, influencing the range of microbial molecules that engage with the host immune system [161].

Besides phages, the gut virome includes eukaryotic viruses that infect human cells, such as rotaviruses, noroviruses, adenoviruses, astroviruses, enteroviruses, and many others [163]. In healthy individuals, the majority of these infections are asymptomatic or cause only mild, short-lived illness. A study by Kernbauer et al. (2014) [164] showed that an innocuous murine norovirus infection in germ-free mice was able to restore intestinal immune functions typically absent in germ-free animals. Germ-free mice have undeveloped gut immunity, but when these mice were mono-colonized with mouse norovirus (MNV), many of their intestinal immune defects were corrected–the virus effectively substituted for the “education” that commensal bacteria typically deliver [164]. This finding introduced the idea of “viral commensalism”: certain viruses can function as commensal-like agents that modulate the baseline immune state [164]. The presence of a harmless virus, mainly through shaping the bacterial population, keeps the immune system vigilant, maintaining structures such as gut-associated lymphoid tissue and priming innate interferon pathways, all without inducing disease symptoms [162]. Constantly, the body is exposed to dietary and environmental viruses. The presence of viral particles in the stool of most healthy individuals indicates that the immune system is consistently detecting and handling these exposures. Table 3 presents current evidence on viral influences in immunotherapy—spanning gut virome diversity, blood anellovirus burden, oncolytic virus combinations, latent virus reactivation, and bacteriophages—and outlines where associations with ICI efficacy or toxicity are established versus still emerging.

### 6.2. Virome Alterations in Cancer and Therapy: Lessons from Virome Depletion

Evidence for the direct influence of the virome on anti-cancer immunity came from a recent mouse study examining how disrupting the virome impacts tumor treatment [165]. Researchers treated tumor-bearing mice with a combination of antiviral drugs to broadly deplete the gut virome and then assessed the outcome of chemotherapy (5-fluorouracil) on colorectal cancer [165]. The antiviral cocktail substantially reduced both DNA and RNA viruses in the mice’s guts, without killing bacteria. This resulted in faster tumor growth, poorer survival and reduced efficacy of chemotherapy compared to control mice with an intact virome [165]. The antiviral-treated mice had fewer dendritic cells and CD8^+^ T-cells within tumors, suggesting an impaired immune response [165]. After the researchers restored the gut virome by conducting a fecal microbiota transplant (FMT) from normal mice into the antiviral-treated mice, the anti-tumor immunity was rescued, and tumor management improved [165]. By investigating the mechanism, they discovered that antiviral treatment had specifically downregulated the TLR3–IRF3–IFN-β pathway in both the gut and tumor microenvironment [165]. In normal mice, a degree of TLR3 signaling likely occurs due to the virome during small episodes of viral replication or release of phage dsDNA/RNA fragments, resulting in the synthesis of IFN-β and chemokines that help recruit dendritic cells and T cells to the tumor. The researchers confirmed this by administering a synthetic TLR3 agonist (polyinosinic: polycytidylic acid, poly(I: C)) to virome-depleted mice, which effectively mimics viral dsRNA. Remarkably, poly(I: C) treatment restored dendritic cell and CD8^+^ T-cell infiltration into tumors and enhanced the efficacy of 5-FU chemotherapy in those mice [165]. This highlights that the immune dysfunction was due to the absence of viral-sensing stimulation, and reintroducing that signal restored anti-tumor immunity. Even though this particular study was in the context of chemotherapy, the principles are likely to extend to immunotherapy with ICIs [33].

### 6.3. Clinical Observations: Virome and Immune Checkpoint Inhibitor Therapy

Direct clinical data regarding the virome’s influence on cancer immunotherapy remains limited, but a few points are worth noting. One attempt to find a virome-based biomarker involved anelloviruses, which are ubiquitous, non-pathogenic DNA viruses that typically rise in titer when the immune system is suppressed. Pescarmona et al. (2021) [166] monitored torque teno virus (TTV) levels present in the blood of advanced melanoma patients receiving anti–PD-1 treatment, reasoning that TTV levels could indicate the degree of immune competence, since elevated TTV implies weaker immune control. However, they found no significant difference in TTV viral load between responders and non-responders, and anti–PD-1 therapy did not consistently alter TTV levels [166]. Prior to treatment, TTV counts in melanoma patients were similar to those of healthy individuals, and during therapy, some patients experienced slight TTV rises or falls regardless of outcome [166]. This negative result suggests that a single plasma virus titer is unlikely to represent an informative biomarker for ICI effectiveness. The virome’s influence might be more complex–potentially involving tissue-localized effects or the combined presence of many virus types. It also shows that not every virus modulates immunity in a way that affects tumor responses. In immunocompetent cancer patients on PD-1 blockers, the immune system is not globally suppressed (in fact, it is activated), so it is perhaps unsurprising that TTV, a marker of severe immunosuppression, was not relevant.

Another aspect of the virome’s relevance lies in immune-related adverse events (irAEs) from ICIs. Evidence suggests that latent viruses can be “unleashed” when checkpoints are blocked, resulting in tissue damage [167,168,169,170,171,172]. A notable example comes from ICI-induced hepatitis: A 2021 study conducted by Hutchinson et al. examined melanoma patients who developed hepatitis on combined anti–PD-1 + anti–CTLA-4 therapy. They found that these patients frequently had an expansion of effector memory CD4^+^ T-cells specific for viruses like Epstein–Barr virus (EBV) or cytomegalovirus (CMV) [173]. Checkpoint blockade not only reactivated T cells against tumor antigens but also memory T cells targeting latent viral antigens, which then caused collateral liver damage. They also observed a seasonal timing–patients with pre-existing high CMV antibody titers and expanded CMV-specific T cells were more prone to hepatitis, suggesting subclinical CMV reactivation in the setting of metastatic disease [173]. Importantly, patients identified to be at risk who received PD-1 monotherapy or prophylactic anti-CMV medication had reduced incidence of hepatitis compared to those given full PD-1+CTLA-4 without preventive measures [173]. This study highlights how the virome can influence toxicity: the loss of PD-1-mediated tolerance enabled a “virus-specific immunopathology” to emerge. Similar mechanisms might underlie other irAEs–some cases of checkpoint-induced encephalitis have been linked to T cells targeting obscure viruses or even gut microbes cross-reacting with brain tissue [174]. Clinicians do sometimes check for viral triggers–interestingly, CMV colitis can flare in ICI patients on steroids, and distinguishing CMV colitis from autoimmune colitis is important for choosing the appropriate treatment [167,169,171]. Enhanced antiviral therapy could be a key strategy to ensure the safe administration of ICIs in infected patients [175].

On the other hand, chronic viruses might sometimes assist anti-tumor immunity. A multicenter study found that cancer patients, excluding those with liver cancer, who happened to have chronic hepatitis B virus (HBV) infection responded better to anti–PD-1 therapy compared to their HBV-negative counterparts [176]. These HBV+ patients had improved objective response rates and extended survival on immunotherapy [176]. Immune profiling indicated that HBV infection had led to a higher baseline frequency of exhausted CD8^+^ T cells (HBV-specific T cells) in these patients, which were reactivated with PD-1 blockade and contributed to anti-tumor effects [176]. This finding is quite intriguing–it implies that not all chronic viral infections are detrimental in cancer therapy. On the contrary, some might create an immune environment that is actually more responsive to checkpoint inhibition.

The concept of using viruses for cancer therapy is not new: oncolytic viruses are an active area of treatment development. T-VEC (talimogene laherparepvec), an engineered herpes simplex virus, is approved for treating melanoma–it infects and lyses tumor cells while promoting local inflammation. Additional oncolytic viruses are currently undergoing trials for various tumors [177,178]. The effectiveness of these approaches further highlights that viruses can serve as potent tools to provoke an immune response against cancers. They essentially convert “cold” tumors into “hot” ones by releasing tumor antigens in the context of viral PAMPs and cell death.

The gut virome, although not intentionally introduced, might perform a mini oncolytic-like function if viral replication occasionally leads to bursts of interferon and cell debris release in the gut, systemically enhancing immune vigilance. However, most of the virome’s impact on immunity likely comes via indirect routes through bacteria [162]. There is evidence in mouse model studies that phage populations shift during cancer progression and in response to treatments [162]. However, distinguishing cause from effect is challenging in vivo due to the complexity of phage dynamics. This area will benefit from thorough preclinical studies.

**Table 3:** Gut Virome and viral factors associated with ICI outcomes.

Viral Factor	Association with ICI Outcomes	Key References
High gut virome diversity	No consistent association with ↑ or ↓ ICI efficacy in humans. Predictive value unclear.	[32,33,146,159]
Blood anellovirus (TTV) load	No significant association with ICI response (melanoma cohort).	[166]
Oncolytic virus therapy (e.g., T-VEC)	↑ ICI efficacy when combined is synergistic tumor immune activation.	[177,178,179,180]
Latent virus reactivation (HBV, EBV, CMV)	↑ irAEs (e.g., hepatitis, myocarditis); PD-1 blockade may trigger viral reactivation.	[4,167,168,169,170,171,172,181,182,183]
Bacteriophages	Indirect effect via bacterial modulation; potential ↓ efficacy or ↑ toxicity if dysbiosis-promoting (evidence preliminary).	[25,155,184,185]

↑ = increased ↓ = decreased.

### 6.4. Section Summary and Key Take-Home Messages

The gut virome influences host immunity both directly, via innate immune sensing pathways such as TLR3–IFN signaling, and indirectly, through modulation of bacterial communities. While clear human virome-based biomarkers for ICI response have not yet been established, preclinical data indicate that virome depletion impairs anti-tumor immunity. Moreover, latent viral reactivation may contribute to immune-related toxicities. These findings position the virome as an underexplored but potentially critical regulator of immunotherapy outcomes.

## 7. Modulation Strategies Targeting the Multi-Kingdom Microbiome in ICI Therapy

### 7.1. Dietary Modulation

#### 7.1.1. High-Fiber and Plant-Rich Diets

Diet significantly influences the composition of the gut microbiota, as shown in Table 4. Recent clinical studies show that high-fiber and plant-rich diets are associated with better ICI outcomes [186], while some other dietary patterns may have negative effects. Observational clinical studies consistently associate high-fiber and plant-rich dietary patterns with improved ICI outcomes. In melanoma patients receiving anti–PD-1 therapy, consumption of ≥20 g/day of dietary fiber correlated with significantly longer progression-free survival (PFS), with each additional 5 g/day associated with a 30% reduction in risk of progression or death [186]. Notably, high-fiber intake combined with avoidance of over-the-counter probiotics was associated with the most favorable outcomes [186]. In the same study, patients on high-fiber diets who avoided probiotic supplements responded best to ICI, while those taking over-the-counter probiotics experienced worse outcomes [186]. A separate cohort study reported that high plant-based food consumption was linked to improved anti–PD-1 responses, while high dairy intake correlated with inferior outcomes [187]. Daily bowel movements—likely reflecting higher fiber intake and improved gut motility—were also associated with better therapeutic responses [187].

Traditional dietary patterns such as the Mediterranean diet have been proposed as supportive measures during immunotherapy [78,188]. These diets promote short-chain fatty acid (SCFA) production, which has been correlated with improved anti–PD-1 outcomes [78].

#### 7.1.2. Mechanistic Insights from Preclinical Models

In mouse models, high-fiber diets increase SCFA-producing taxa (e.g., *Ruminococcaceae*, *Faecalibacterium*) and enhance CD8^+^ T-cell effector function. SCFAs activate GPCR41/43 signaling on immune cells and modulate dendritic cell and T-cell metabolism, providing a plausible mechanistic link between diet and ICI efficacy [78].

##### Diets Potentially Associated with Inferior Outcomes

In contrast, diets high in simple sugars and fats, such as the “Western” diet, may promote microbial metabolites that are less favorable or potentially immunosuppressive [69,189,190,191]. Excessive sugar also supports *Candida albicans*, a commensal yeast that can overgrow with high-carb diets and antibiotic use, potentially skewing immunity toward inflammatory Th17 responses [130]. Therefore, a balanced diet that is low in refined carbohydrates may help in keeping the gut mycobiome in check.

Fasting, Caloric Restriction, and Ketogenic Diets. Fasting and ketogenic diets have been taken into consideration as well [98,188]. Intermittent fasting or calorie-restricted eating can positively alter the microbiome and immune profile: a recent systematic review found that pairing ICIs with an intermittent fasting or fasting-mimicking diet enhanced tumor control in mouse models and was associated with higher levels of SCFA-producing bacteria in both mice and patients [98]. These bacteria are known for their immunomodulatory metabolites and have shown a positive correlation with objective responses in patients [98,192,193,194]. Ketogenic diets, on the other hand, may result in a reduced fiber intake and potentially decrease microbiota diversity and SCFA production.

##### Human Data

Human evidence remains limited. Systematic reviews suggest that fasting-mimicking approaches may correlate with improved microbiome composition and immune markers [98], but randomized trials in ICI-treated patients are lacking.

Ketogenic diets may reduce fiber intake and SCFA production, potentially decreasing microbiota diversity. Although sometimes explored in oncology for metabolic reasons, caution is warranted given limited immunotherapy-specific data [188,195].

Ongoing trials are investigating high-fiber and Mediterranean dietary interventions in ICI-treated patients [195,196,197]. In clinical practice, nutritional guidance may become a standard component of immunotherapy care.

**Table 4:** Key microbial species associated with dietary interventions that may impact ICI responses.

Dietary Intervention	Microbiome/Immune Association	Key References
High-fiber, plant-rich diet	↑ SCFA-producers (*Faecalibacterium*, *Bifidobacterium*, *Akkermansia*, Ruminococcaceae); ↓ *Candida* overgrowth; ↑ ICI efficacy (observational).	[78,186,198,199,200,201,202,203]
Mediterranean diet	↑ Microbial diversity; ↑ beneficial taxa; ↓ inflammatory markers; potential ↑ ICI efficacy (associative).	[78,98,190,198,202,204,205,206,207,208]
Western diet	↓ SCFA-producers; ↑ bile-tolerant/pathobiont taxa; ↑ *Candida* spp.; potential ↓ ICI efficacy; ↑ inflammatory tone.	[69,189,190,191,202,209,210,211,212]
Ketogenic/very low-carb diet	↓ Fiber-dependent commensals; ↓ SCFA production; ↑ protein/fat metabolizers; effect on ICI efficacy unclear.	[202,213,214,215]
Intermittent fasting/caloric restriction	↑ *Akkermansia* and Ruminococcaceae; ↑ SCFA output (refeeding phase); potential ↑ ICI efficacy (preclinical/early data).	[98,192,193,194,212,216,217]

↑ = increased ↓ = decreased.

### 7.2. Prebiotics and Supplements

#### 7.2.1. Prebiotics

##### Mechanistic and Preclinical Data

Prebiotics are nutrients, usually fibers or oligosaccharides, that specifically support healthy gut microbes. Supplementing cancer patients’ diets with prebiotics might enhance ICI efficacy by increasing beneficial bacteria and their metabolites, as shown in Table 5. Common prebiotics include inulin and fructo-oligosaccharides (FOS), galacto-oligosaccharides, resistant starch, and pectins. Resistant starch supplementation is known to enhance the growth of starch-degrading *Ruminococcaceae* and related fermenters, increasing butyrate and SCAFs production in the colon [218]. Inulin/FOS promote growth of *Bifidobacterium* species, which have been associated with improved responses in melanoma and other cancers [98,219,220]. Another example is polyphenols in green tea, pomegranate, and berries which are metabolized by gut bacteria into bioactive compounds that may improve anti-tumor immunity [18,221]. These can increase the prevalence of beneficial taxa like *Lactobacillus* and *Bifidobacterium*, simultaneously suppressing pathogenic bacteria and fungi–polyphenols have mild antimicrobial properties that spare commensals [18,69,198].

##### Human Evidence

Human data primarily demonstrate microbiome shifts rather than direct improvements in ICI outcomes. No large, randomized trials have yet evaluated prebiotics specifically in ICI-treated patients. Thus, prebiotics and supplements are mechanistically promising but clinically unvalidated in this setting.

#### 7.2.2. Postbiotics and Supplements

Postbiotics include microbial metabolites or components administered directly and can be considered as dietary supplements. Butyrate or its derivatives could potentially be administered to replicate the effect of a fiber-rich microbiome in stimulating anti-tumor T cells and protecting the gut lining [144,222,223]. β-glucans, complex polysaccharides derived from fungal cell walls, are another supplement currently being studied [134]. When taken orally, particulate yeast β-glucan can be taken up by gut phagocytes and ultimately prime neutrophils in the bone marrow to destroy tumor cells more effectively, while also activating dendritic cells [134,136,224]. These immunostimulatory polysaccharides function primarily as oral adjuvants, triggering PRRs and promoting a Th1-dominated, anti-tumor immune response [69]. In a similar manner, certain bacterial TLR agonists may be regarded as “postbiotic” immune supplements–though these are more often delivered as drugs or intratumoral agents rather than oral supplements. Another important “supplement” to consider is vitamin D. Adequate vitamin D levels have been correlated with a favorable microbiome environment and immune response. A study focused on advanced melanoma patients revealed that individuals with adequate 25(OH)D levels (from supplementation or sunlight) during anti–PD-1 therapy had significantly higher objective response rates (56% vs. 36% ORR) and longer PFS (11.25 vs. 5.75 months) compared to vitamin D–deficient patients [225]. Although that study did not directly analyze the microbiome, separate research has noted that vitamin D can shape the gut microbiota–patients low in vitamin D had lower abundance of *Ruminococcaceae* and *Lachnospiraceae*, which increased following vitamin D repletion [78,226]. The retrospective analysis found even better ICI outcomes and fewer colitis events in patients taking vitamin D supplements [225,226]. Given vitamin D’s role in mucosal immunity and maintaining barrier integrity, ensuring cancer patients are not deficient could be a simple step to potentially reduce dysbiosis and inflammation.

**Table 5:** Key microbes that are promoted by prebiotics and related supplements.

Prebiotic/Supplement	Microbiome/Immune Association	Key References
Inulin, FOS	↑ *Bifidobacterium*, ↑ *Lactobacillus*, ↑ *Faecalibacterium*; ↑ SCFA production; potential ↑ ICI efficacy (associative).	[186,219,220,227,228]
Resistant starch	↑ *Ruminococcus bromii*, ↑ *Eubacterium rectale*; ↑ SCFAs; ↑ CD8^+^ T-cell function (mechanistic).	[78,155,218,229]
Polyphenols	↑ *Lactobacillus*, ↑ *Akkermansia*; ↓ Enterobacteriaceae; ↓ tumor growth (preclinical).	[69,202,203,221,230]
Vitamin D	↑ Lachnospiraceae, ↑ Ruminococcaceae; associated with ↑ ICI response and ↓ colitis risk (retrospective).	[78,100,226]
Butyrate (postbiotic)	Direct SCFA supplementation; ↑ CD8^+^ memory; ↑ immune regulation (context-dependent).	[69,155,231,232]
β-glucan	Immune activation via PRRs; ↑ Th1 response; ↑ ICI efficacy (preclinical synergy).	[148,149,150,151,152,155,224]

↑ = increased ↓ = decreased.

SCFAs are key players since they are messengers between microbiota and immune cells. Systemic butyrate and propionate levels have been correlated with better ICI responses–patients with higher serum butyrate had better anti–PD-1 outcomes in cancer studies [78,222]. SCFAs can bind to GPR41/43 on immune cells [78]; butyrate, in particular, influences dendritic cell and T-cell differentiation by promoting IL-10 and Tregs locally, while also enhancing CD8^+^ memory and IFN-γ production systemically at low concentrations [69]. Therapeutically, can be provided through oral resistant starch (prebiotic) or encapsulated butyrate. There is also interest in using butyrate enemas for the treatment of checkpoint-induced colitis while maintaining anti-tumor immunity.

Overall, postbiotic strategies offer a strong mechanistic rationale, with emerging but still limited clinical validation.

### 7.3. Probiotics and “Mycobiotics”

#### 7.3.1. Conventional Probiotics

Probiotics are live microorganisms administered for health advantages. In cancer immunotherapy, probiotics have a complex role, yet to be fully understood [86,233,234,235]. The majority of probiotics available on the market contain strains of *Lactobacillus* or *Bifidobacterium* that are generally beneficial for gut health [100]. Despite that, unregulated use of generic probiotics has not demonstrated a clear benefit in ICI-treated patients [235]. An observational study found that melanoma patients taking over-the-counter probiotics had worse responses to PD-1 blockade, particularly if their dietary fiber intake was low [186]. One hypothesis is that these standard probiotics might not integrate well or could even reduce the diversity of the native microbiome if used carelessly. Overwhelming the gut with large amounts of exogenous *Lactobacillus* could outcompete certain commensals that are actually more crucial for anti-cancer immunity (such as *Akkermansia* or *Faecalibacterium*) or simply may not address the specific deficiencies of an individual’s microbiome [186].

#### 7.3.2. Next-Generation Probiotics and Defined Consortia

Routine, non-personalized probiotic use cannot currently be recommended in ICI-treated patients. The focus is therefore shifting to next-generation probiotics–choosing commensal bacteria recognized by research as enhancing ICI efficacy and delivering them in a targeted way. Consortia of multiple selected commensals-“VE800”, a defined 11-strain mix by Vedanta Biosciences used in NCT04208958 clinical trial, and MB097 (nine different species of gut commensal bacteria, all linked to positive CPI response in multiple clinical studies) used in NCT06540391 clinical trial. Early results from these trials are pending, but they represent an initial shift to a rational, personalized probiotic approach.

#### 7.3.3. Mycobiotics and Engineered Yeasts

Beyond bacteria, probiotic fungi, also referred to as “mycobiotics”, are an emerging concept. The best-known example is *Saccharomyces boulardii*, a non-pathogenic yeast commonly used to prevent or treat antibiotic-associated diarrhea and Clostridioides difficile infection [236]. *S. boulardii* modulates gut immunity and releases enzymes that neutralize bacterial toxins [237]. Supporting this concept, a recent proof-of-principle study engineered *S. cerevisiae* var. *boulardii* to secrete miniature anti-PD-L1 proteins (Sb_haPD-1), showing that orally delivered yeast can transiently transit the gut, secrete functional ICIs in situ, be well tolerated, shift gut bacterial communities, reduce Treg prevalence in mesenteric nodes, and significantly lower distal small-intestinal tumor burden in an ICI-refractory colorectal cancer mouse model [238]. Although *S. boulardii* has not been specifically studied in ICI patients, it is speculated that it could help maintain microbiome balance during therapy or antibiotic use and possibly lower the risk of checkpoint-induced colitis by outcompeting opportunistic *Candida.*

Personalized probiotic approaches will probably be necessary–using stool sequencing to identify which key beneficial microbes a patient lacks, followed by supplementing those. Irresponsible use of one-size-fits-all probiotics should be replaced by this tailored approach, which promises improved results as summarized in Table 6.

**Table 6:** Examples of probiotic organisms and their potential relevance alongside immunotherapy treatment.

Probiotic Organism	Association with ICI Outcomes	Key References
*Akkermansia muciniphila*	↑ ICI efficacy (responders enriched); ↑ DC activation and mucosal immunity; under therapeutic development.	[24,95,98,117,239,240]
*Faecalibacterium prausnitzii*	↑ ICI efficacy; ↑ SCFA production; potential ↓ colitis risk; formulation challenges (strict anaerobe).	[186]
*Bifidobacterium longum*/*B. breve*	↑ Anti–PD-L1 efficacy (preclinical); ↑ DC activation and CD8^+^ infiltration; human association supportive.	[21,98,100,101]
*Bacteroides fragilis*	↑ CTLA-4 efficacy (preclinical); Th1 induction; potential inclusion in defined consortia (caution as mono-strain).	[105,155]
*Saccharomyces boulardii*	Potential microbiome stabilization; engineered strains → ↑ ICI efficacy (preclinical); clinical data in ICI limited.	[236,237,238]

↑ = increased ↓ = decreased.

### 7.4. Fecal Microbiota Transplantation (FMT)

#### 7.4.1. Preclinical Evidence

Fecal microbiota transplantation–the transfer of stool from a healthy donor to a patient–is the most straightforward method to modify the entire gut microbiome. FMT is already an established therapy for refractory *C. difficile* infection [241], and now it is being tested as an immunotherapy adjunct [15,242,243]. Evidence from both animal and human studies indicates that germ-free or antibiotic-treated mice have diminished responses to ICIs, which can be restored by transplanting feces from responder patients [242,244,245].

##### Human Interventional Trials

In humans, important trials in 2021 offered strong proof that FMT can influence ICI outcomes [26,28]. In melanoma patients unresponsive to anti–PD-1, FMT from elite responders combined with resuming anti–PD-1 therapy led to clinical responses in a subset of recipients [26,27,28]. A certain percentage of previously refractory patients experienced tumor regression or disease stabilization following FMT plus pembrolizumab, presumably due to engraftment of immunomodulatory microbes from the donors [26]. Bacterial taxa, which may be responsible for FMT success in ICI-treated patients, are summarized in Table 7.

Overall, FMT is in its initial phase within oncology, but the early successes in melanoma have sparked hope [75,245]. Larger trials are underway to determine if FMT can consistently rescue patients who do not respond to ICIs [242,246]. If validated, FMT or next-gen microbiome products inspired by FMT could become part of the immunotherapy arsenal.

**Table 7:** Key species involved in FMT success in the context of immunotherapy.

FMT Donor Microbe/Feature	Association After Engraftment	Key References
*Akkermansia muciniphila*	Engraftment → ↑ PD-1 sensitivity; ↑ T-cell activation; associated with ICI re-sensitization.	[24,27,94,95,242,245,247]
*Faecalibacterium prausnitzii*	↑ Post-FMT abundance → ↑ ICI efficacy; ↓ colitis risk; ↑ SCFA production.	[25,26,242]
*Ruminococcaceae* spp.	↑ Diversity restoration; ↑ SCFA production; associated with responder phenotype.	[27,242,247]
*Bacteroides* spp.	Context-dependent; associated with favorable immune modulation post-FMT; requires monitoring for overabundance.	[242,247]
High overall microbial diversity	↑ ICI efficacy; ecosystem restoration; hallmark of successful engraftment.	[27,242,248]
*Candida* spp. (*mycobiome transfer*)	Role unclear; fungal transfer occurs; no clear ↓ efficacy signal in responders; requires donor screening.	[26,27,156]

↑ = increased ↓ = decreased.

FMT’s broad mechanism–resetting the gut ecosystem–could influence not just efficacy but also toxicity [111,122]. Interest exists in using FMT to address immune-related colitis, with some case reports showing FMT can help ICI-associated colitis and steroid-refractory colitis in ICI patients by reintroducing regulatory microbes [93,249,250]. This dual benefit, enhancing anti-tumor immunity while healing the gut lining, would make microbiome modulation quite unique in therapy.

**Figure 2:**
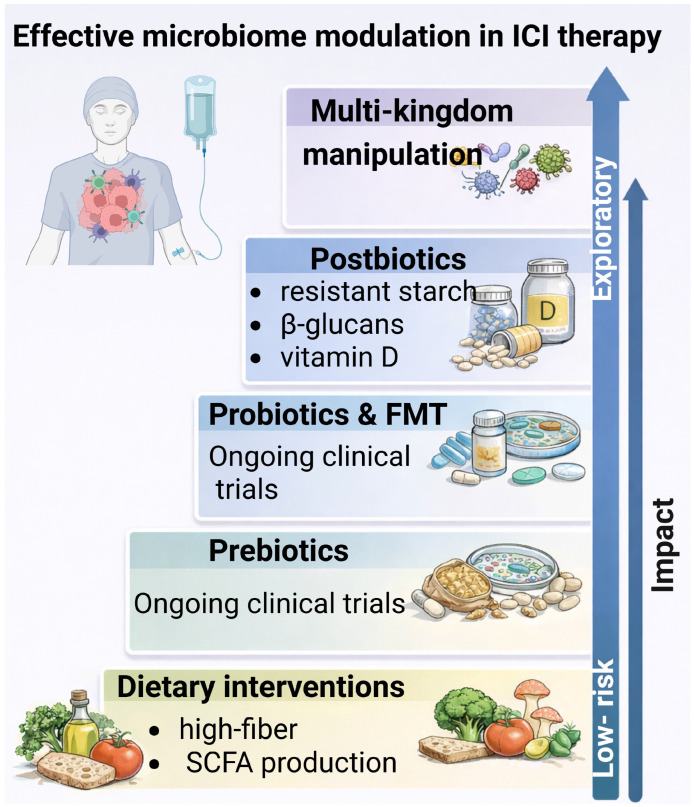
Translational hierarchy of microbiome modulation strategies in immune checkpoint inhibitor (ICI) therapy. Schematic overview of microbiome-targeted interventions organized along a conceptual continuum from low-risk, broadly applicable approaches to higher-impact, ecosystem-level manipulation. Dietary interventions (e.g., high-fiber intake promoting SCFA production) represent foundational, low-risk strategies associated with improved ICI responsiveness. Prebiotic supplementation further supports beneficial microbial taxa and metabolic outputs. Probiotics, defined microbial consortia, and FMT aim to restore specific taxa linked to therapeutic sensitivity and are currently under clinical evaluation. Postbiotic approaches (e.g., resistant starch–derived SCFAs, β-glucans, vitamin D) directly modulate immune–microbial signaling pathways. At the highest level of intervention, multi-kingdom manipulation targets bacterial, fungal, and viral communities simultaneously to reprogram systemic immune tone. The vertical axis reflects increasing biological impact and translational complexity, highlighting the progressive shift from supportive modulation to ecosystem reconstitution to optimize ICI efficacy and safety.

To provide a conceptual framework for the translational landscape of microbiome-targeted interventions in immune checkpoint inhibitor (ICI) therapy, we summarize current modulation strategies in Figure 2. These approaches span a continuum from broadly applicable, low-risk dietary interventions to advanced ecosystem-level manipulation of the multi-kingdom microbiome. Importantly, strategies differ not only in biological impact but also in translational maturity, ranging from observational human associations to early interventional trials and emerging engineered microbial therapeutics. This hierarchy underscores the progressive shift from supportive metabolic modulation toward targeted reconstitution of bacterial, fungal, and viral networks aimed at optimizing anti-tumor immunity while minimizing immune-related toxicity.

### 7.5. Toward Personalized Microbiome Modulation in ICI Therapy

Although the microbiome modulation strategies discussed above are supported by increasing mechanistic and clinical evidence, most proposed regulatory approaches are currently derived from population-level associations rather than individualized therapeutic frameworks. Given the heterogeneity of tumor types, host genetic background, immune baseline status, prior antibiotic exposure, and baseline multi-kingdom microbiome composition, uniform application of these interventions may not be appropriate. Therefore, translation of microbiome-directed strategies into clinical practice requires structured patient stratification and a clearer definition of the specific clinical scenarios in which each intervention may be relevant.

Evidence indicates that microbiome signatures associated with ICI responsiveness differ according to cancer type. In melanoma, enrichment of *Akkermansia muciniphila*, *Faecalibacterium prausnitzii*, and *Bifidobacterium* spp. has been consistently associated with favorable anti–PD-1 responses [24,95,98,186,239,240], and the most advanced interventional data derive from melanoma cohorts. Notably, phase I/II trials have demonstrated that fecal microbiota transplantation (FMT) from ICI responders can re-sensitize a subset of anti–PD-1–refractory melanoma patients [26,27,28,242], providing proof-of-concept for ecosystem-level modulation in a defined clinical context. In contrast, data in non-small cell lung cancer and renal cell carcinoma remain more heterogeneous, and antibiotic exposure appears particularly detrimental in these settings [69,189,190,191], suggesting that microbiome-preserving strategies may be especially important during peri-immunotherapy management. In hepatocellular carcinoma, preliminary evidence implicates fungal diversity as a potential predictor of response [156], indicating that multi-kingdom profiling may be clinically informative in selected tumor types. These observations collectively argue against universal microbiome interventions and instead support tumor-type–adapted regulatory strategies.

Baseline microbiome configuration may further inform clinical decision-making. Patients presenting with low-diversity, pathobiont-enriched microbiomes—particularly following recent antibiotic exposure—have been associated with inferior ICI outcomes [242,243,244,245]. In such scenarios, structured dietary fiber enrichment [186,187], prebiotic supplementation targeting SCFA-producing taxa [218,219,220], or investigational FMT approaches [26,27,28] may be rationally considered within clinical trial settings. Conversely, patients with microbiomes already enriched in immunostimulatory taxa may require cautious modulation, as excessive immune activation could increase susceptibility to immune-related adverse events. Fungal dysbiosis characterized by *Candida* overrepresentation [130,156] may warrant targeted dietary carbohydrate moderation or antifungal stewardship rather than empiric probiotic use. These distinctions highlight the potential clinical utility of baseline multi-kingdom microbiome profiling prior to implementing regulatory interventions.

Host-related factors also influence the clinical applicability of microbiome modulation. Variability in pattern recognition receptor signaling, vitamin D receptor function, and baseline T-cell activation states may determine whether microbial-derived metabolites translate into beneficial anti-tumor immunity or heightened inflammatory toxicity [69,78,226]. Notably, vitamin D sufficiency has been associated with improved objective response rates and progression-free survival in anti–PD-1–treated melanoma patients [225], alongside favorable microbiome shifts [226]. Given its safety profile and low cost, correction of vitamin D deficiency represents one of the few microbiome-related interventions that may already be pragmatically incorporated into supportive oncology care, pending prospective validation.

Importantly, the clinical maturity of available interventions differs substantially. High-fiber and Mediterranean-style dietary patterns are supported by observational human data and mechanistic plausibility and may be incorporated into nutritional counseling during immunotherapy [78,186,197]. Prebiotics and polyphenols demonstrate reproducible microbiome modulation but lack randomized evidence demonstrating improved ICI outcomes [18,218,219,220,221], and should therefore be considered investigational in this setting. β-glucans have shown synergistic effects with checkpoint blockade in preclinical models [148,149,150,151,152] and are currently being explored as adjuvant strategies in early-phase trials. Defined microbial consortia, including rationally selected bacterial strains, are under active clinical investigation but remain experimental. FMT has demonstrated proof-of-concept efficacy in anti–PD-1–refractory melanoma [26,27,28], yet requires standardized donor screening, long-term safety monitoring, and regulatory harmonization before broader implementation [242,246]. Engineered microbial platforms represent promising translational innovations but remain at the preclinical stage [238].

Collectively, these data support the development of a structured precision framework for microbiome modulation in immuno-oncology. Such a framework would integrate baseline multi-kingdom profiling, tumor-type-specific evidence, host immunologic context, and clearly defined clinical endpoints, while restricting higher-risk ecosystem-level interventions to controlled clinical trials. Transitioning from generalized population-based recommendations toward stratified, indication-specific regulatory strategies may enhance therapeutic benefit while minimizing unintended immune dysregulation, thereby aligning microbiome-directed interventions with the principles of precision oncology.

## 8. Integrative Multi-Kingdom Mechanisms and Emerging Hypotheses

The gut microbiome should not be conceptualized as separate bacterial, fungal, and viral compartments acting independently, but rather as a dynamically interconnected ecological system whose members influence one another and collectively shape host immunity. Although bacterial components have been most extensively studied in the context of ICI therapy, emerging evidence indicates that cross-kingdom interactions critically modulate immune tone, treatment responsiveness, and toxicity risk. These interactions are mediated through convergent innate immune sensing pathways, metabolic exchange, ecological competition, bacteriophage-driven population control, and systemic immune signaling.

A central integrative mechanism involves overlapping innate immune recognition pathways. Bacterial products such as lipopolysaccharide and peptidoglycans, fungal β-glucans, and viral nucleic acids activate pattern recognition receptors, including Toll-like receptors (TLRs), Dectin-1, and RIG-I-like receptors. These pathways converge on NF-κB and type I interferon signaling cascades, which regulate dendritic cell maturation, antigen presentation, and CD8^+^ T-cell priming—core processes underlying ICI responsiveness. Baseline variation in tonic stimulation through these receptors may establish distinct immune “set points,” thereby influencing both sensitivity to checkpoint blockade and susceptibility to immune-related adverse events (irAEs).

Cross-kingdom ecological interactions further modulate metabolite production and immune signaling. Fungal expansion, particularly following antibiotic exposure, can alter bacterial community structure and reduce the abundance of short-chain fatty acid (SCFA)-producing commensals, indirectly attenuating regulatory and metabolic support for effector T cells. Conversely, certain fermentative yeasts may contribute to SCFA pools, reinforcing epithelial integrity and immune homeostasis. Fungal-derived metabolites—including ethanol, organic acids, and quorum-sensing molecules such as farnesol—can influence bacterial biofilm formation and virulence programs, reshaping the broader microbial ecosystem. In parallel, fungal cell wall components such as β-glucans activate Dectin-1–mediated pathways that modulate antimicrobial peptide production and mucosal cytokine gradients, secondarily altering bacterial colonization patterns relevant to ICI response.

Bacteriophages represent another critical regulatory layer within this ecosystem. Through lytic activity, phages can selectively reduce dominant bacterial populations, limiting pathobiont overgrowth and preserving microbial diversity. Conversely, prophage activation under inflammatory stress may destabilize beneficial communities. Phages also facilitate horizontal gene transfer, influencing bacterial metabolic capacity, toxin production, and immunogenic potential. Importantly, phage-driven modulation of SCFA-producing bacteria or mucin-degrading taxa may indirectly shape systemic immune priming. In addition, phage-derived nucleic acids can stimulate innate immune receptors such as TLR3 and TLR9, reinforcing type I interferon pathways that are central to dendritic cell activation and cytotoxic T-cell responses in the context of checkpoint blockade.

Collectively, these multi-kingdom interactions suggest that immune activation and toxicity may represent different thresholds along a shared inflammatory continuum. Microbiome configurations that strongly prime dendritic cells and effector T cells may enhance tumor control while simultaneously lowering mucosal tolerance, thereby increasing the risk of irAEs. This framework may help explain the partial overlap between microbial signatures associated with ICI efficacy and those linked to immune-mediated toxicity.

Based on these integrative observations, several testable hypotheses emerge. Synergistic innate priming may occur when fungal β-glucans and SCFA-producing bacteria jointly enhance dendritic cell maturation and antigen presentation, amplifying ICI responsiveness. Phage-mediated ecosystem regulation may indirectly influence therapeutic efficacy by selectively targeting bacterial taxa associated with immune suppression or activation. Baseline gut virome composition may modulate systemic type I interferon tone, shaping both response probability and toxicity risk. Finally, microbiome configurations that optimize anti-tumor immunity may simultaneously reduce the threshold for autoimmunity, reflecting a shared inflammatory axis underlying both benefit and adverse events.

These considerations underscore the need to move beyond single-microbe supplementation strategies toward ecosystem-level modulation guided by multi-kingdom profiling. Integrative ecological network modeling, longitudinal multi-omics analyses, and functional validation studies will be required to disentangle synergistic and antagonistic interactions across bacterial, fungal, and viral communities. Such an approach may ultimately enable more precise calibration of immune activation in ICI therapy, maximizing anti-tumor efficacy while preserving tissue tolerance.

## 9. Methodological and Conceptual Limitations in Multi-Kingdom Microbiome Research

Despite rapid advances in microbiome–oncology research, several important limitations must be acknowledged when interpreting current findings.

First, substantial heterogeneity exists across study populations, including differences in cancer type, disease stage, prior therapies, geographic region, dietary patterns, antibiotic exposure, proton pump inhibitor use, and comorbidities. These variables independently shape microbiome composition and may confound associations with immune checkpoint inhibitor (ICI) outcomes. Tumor-intrinsic factors and host genetic background further complicate the interpretation of microbiome-response relationships.

Second, methodological variability remains a major challenge. Differences in sequencing platforms (16S rRNA gene sequencing versus shotgun metagenomics), fungal ITS amplification strategies, virome enrichment protocols, DNA extraction methods, contamination control, and bioinformatic pipelines limit reproducibility and cross-study comparability. Multi-kingdom analyses are particularly sensitive to technical biases, given the lower biomass and detection challenges associated with fungi and viruses. Viral reference databases remain incomplete, and distinguishing resident viruses from transient dietary or environmental exposures is difficult.

Third, most clinical studies rely primarily on baseline stool sampling, with limited longitudinal assessment. However, microbiome composition is dynamic and may shift substantially during ICI therapy, particularly at the onset of immune-related adverse events (irAEs). Without serial sampling, it is challenging to differentiate predictive microbial signatures from reactive changes secondary to inflammation, corticosteroid treatment, or disease progression.

Fourth, the majority of available human data are observational and associative. Although fecal microbiota transplantation (FMT) trials and selected mechanistic mouse studies provide preliminary causal evidence, large randomized controlled trials validating microbiome-based interventions remain scarce. Furthermore, preclinical models may not fully recapitulate the complexity of human immune regulation or multi-kingdom ecological interactions.

Fifth, current research remains disproportionately focused on bacterial taxa, while the mycobiome and virome are under-characterized. Functional validation of fungal- and phage-mediated mechanisms is limited, and cross-kingdom interaction models are still emerging. The absence of standardized multi-omics integration frameworks further restricts mechanistic insight.

Finally, publication bias toward positive associations may overestimate effect sizes and reinforce non-reproducible microbial “signatures.” Small cohort sizes and limited external validation increase the risk of overfitting predictive models.

Recognizing these limitations is essential to avoid premature clinical implementation of microbiome-based biomarkers or modulation strategies. Rigorous standardization, larger multi-center cohorts, longitudinal multi-omics designs, and mechanistic validation studies will be critical to translating microbiome research into safe and reproducible clinical applications.

## 10. Future Research Directions: Toward Translational Multi-Kingdom Microbiome Modulation

Future research should move beyond the identification of isolated microbial taxa and instead prioritize the characterization of core functional microbiota and ecological networks that directly modulate host immunity. Taxonomic associations, while informative, may not adequately capture the mechanistic complexity underlying ICI responsiveness and toxicity. Functional screening approaches integrating metagenomics, metabolomics, transcriptomics, and immune phenotyping are, therefore, essential to stratify patients according to immunologically relevant microbial signatures rather than relative abundance alone.

A major scientific priority will be the definition of core immune-modulatory microbial networks. Systems biology strategies and ecological network modeling should aim to identify reproducible cross-kingdom clusters—comprising bacterial, fungal, and viral components—that are consistently associated with durable clinical responses or, conversely, with severe immune-related adverse events (irAEs). Such network-based models may provide more robust predictive frameworks than single-organism biomarkers and better reflect the integrated nature of microbiome–immune interactions.

Longitudinal multi-omics monitoring represents another critical direction. Serial sampling of stool, blood, and, where feasible, tumor tissue during ICI therapy will allow discrimination between baseline predictive signatures and secondary microbial shifts that arise as a consequence of immune activation, toxicity, or treatment-related perturbations. This temporal resolution is necessary to distinguish causative microbial drivers from reactive ecological changes and to identify windows of therapeutic opportunity for microbiome modulation.

Mechanistic validation will be required to establish causality. Gnotobiotic mouse models colonized with defined multi-kingdom consortia, as well as human intestinal organoid systems integrated with immune components, provide powerful platforms to dissect specific host–microbe and cross-kingdom pathways. These experimental systems can clarify how defined microbial networks influence dendritic cell maturation, T-cell priming, mucosal barrier integrity, and systemic immune tone in the context of checkpoint blockade.

Future modulation strategies should increasingly adopt rational, targeted approaches rather than broad ecosystem transplantation. While fecal microbiota transplantation has provided proof-of-concept evidence for therapeutic re-sensitization, next-generation strategies may include defined microbial consortia selected for immune-enhancing properties, engineered probiotics capable of delivering checkpoint modulators or immunoregulatory molecules, targeted bacteriophage therapies designed to eliminate pathobionts, and fungal-derived immunomodulators such as β-glucans that selectively amplify innate and adaptive immune responses. Such precision-based interventions may offer greater reproducibility and safety than empiric microbiome replacement.

Importantly, long-term safety and immune equilibrium must remain central considerations. Interventions that enhance anti-tumor immunity may also lower tolerance thresholds and increase the risk of delayed autoimmunity, chronic inflammation, or ecological instability within the gut ecosystem. Therefore, microbiome-based interventions should be accompanied by extended clinical follow-up, immune monitoring, and registry-based surveillance to assess the durability of benefit and late toxicities.

Finally, standardization initiatives are urgently needed to improve reproducibility across studies. International consortia should harmonize sequencing methodologies, bioinformatic pipelines, metadata reporting, and clinical response criteria to facilitate cross-cohort comparison and meta-analytic validation. Establishing standardized frameworks for microbiome data integration will be essential for translating associative findings into clinically actionable tools.

Collectively, these structured research priorities may accelerate the transition from descriptive microbiome associations to mechanistically grounded, safe, and personalized microbiome-based adjuvants in immuno-oncology.

## 11. Conclusions

In conclusion, the gut bacteriome plays a key role in modulating the host immune system’s readiness to fight cancer. Insights gained from bacteria have established the paradigm that manipulating the microbiota could serve as a novel adjunct strategy in oncology–either by predicting which patients are likely to respond to immunotherapy or by actively altering the microbiome to improve outcomes. Current research is extending this investigation to the other constituents of the microbiome. The upcoming challenge is understanding how gut fungi and viruses, which live alongside bacteria, influence the immunological environment in cancer patients receiving ICIs. Early evidence suggests that these, too, may have significant effects on T cell activation status and on which bacteria thrive or decline under therapy. Therefore, while bacteria have been at the forefront of microbiome-cancer research, a holistic view of the “oncobiome” is emerging, promising a deeper and more complete understanding of the host–microbe interactions in cancer immunotherapy.

## Data Availability

No new data were created or analyzed in this study. Data sharing is not applicable to this article.
